# Obesity-induced diet leads to weight gain, systemic metabolic alterations, adipose tissue inflammation, hepatic steatosis, and oxidative stress in gerbils (*Meriones unguiculatus*)

**DOI:** 10.7717/peerj.2967

**Published:** 2017-03-02

**Authors:** Luciana L.A. Ventura, Nathália C.L. Fortes, Helton C. Santiago, Marcelo V. Caliari, Maria A. Gomes, Dirce R. Oliveira

**Affiliations:** 1 Department of Parasitologia/Instituto de Ciências Biológicas, Universidade Federal de Minas Gerais, Belo Horizonte, Minas Gerais, Brazil; 2 Department of Nutrição/Escola de Enfermagem, Universidade Federal de Minas Gerais, Belo Horizonte, Minas Gerais, Brazil; 3 Department of Bioquímica e Imunologia/Instituto de Ciências Biológicas, Universidade Federal de Minas Gerais, Belo Horizonte, Minas Gerais, Brazil; 4 Department of Patologia Geral/Instituto de Ciências Biológicas, Universidade Federal de Minas Gerais, Belo Horizonte, Minas Gerais, Brazil

**Keywords:** Diet-induced obesity, Animal models, Obesity, Lipid metabolism

## Abstract

**Background:**

Nowadays, the number of obese people in the world has reached alarming proportions. During the expansion of adipose tissue, a number of functions such as activation and release of cytokines and hormones may be affected. This leads the body to a pro-inflammatory pattern, which may affect the proper functioning of many tissues. Thus, studying the mechanisms by which obesity induces physiological disorders is necessary, and may be facilitated by the use of animal models, in particular rodents. We sought to characterize the metabolic and adipose tissue changes resulting from a diet rich in fats and simple sugars in gerbils.

**Methods:**

We divided 14 gerbils into two experimental groups that received a diet rich in simple carbohydrates and fats with 5,86 kcal/g (OB, *n* = 7) or a standard diet with 4.15 kcal/g (CT; *n* = 7) for 11 weeks. The animals had free access to water and food. The animal weight and food consumption were measured weekly. Blood, adipose tissue and liver of each animal were collected at the end of experiment. The following parameters were determined: cholesterol (COL), triglycerides (TGL) and glycemia (GLI) in the plasma; cytokines (IL-6, IL-10 and TNF-α) and hormones (adiponectin and leptin) in adipose tissue; activity of superoxide dismutase (SOD) and catalase (CAT), extraction and differentiation of fat and histology in liver.

**Results:**

The consumption of a diet rich in simple carbohydrates and fats led to increased total body weight and increased relative weights of liver and adipose tissue. In addition, we observed increased fasting glucose levels and circulating triglycerides, along with high TNF-α production in adipose tissue and increased total fat, cholesterol and triglyceride contents in the liver, contributing to higher intensity of hepatic steatosis. On the other hand, the animals of this group showed depletion in the enzyme activity of SOD and CAT in the liver, as well as reduction of IL-10 and adiponectin levels in adipose tissue.

**Discussion:**

High intake of saturated fat and simple carbohydrates establish the gerbil as an experimental model for the study of metabolic and hepatic abnormalities resulting from obesity.

## Introduction

In past decades, malnutrition was the main nutritional disorder requiring multi-professional care. Currently, much of the world’s population has experienced the opposite problem, and the number of obese people in the world, has reached alarming proportions. The change in dietary pattern of the world population, making it rich in calories from fats and sugars, associated with physical inactivity, significantly increased prevalence of obesity not only in developed but also in developing countries (WHO, 2016). This fact has drawn much attention since obesity is strongly associated with increased risk of developing metabolic syndrome, characterized by central obesity, insulin resistance, dyslipidemia, increased blood pressure and nonalcoholic fatty liver disease (NAFLD) (Lumeng & Saltiel, 2011).

Adipose tissue is not simply an energy inert deposit, but is a multifunctional organ, that exerts important endocrine and immune functions (Lumeng & Saltiel, 2011; Clemente-Postigo et al., 2011; Gregor & Hotamisligil, 2011). Adipocytes are mainly responsible for the activation and release of substances, such as cytokines IL-6, TNF-*α* and hormones such as leptin and adiponectin (Trujillo et al., 2004; Jung & Choi, 2014). During the expansion of adipose tissue, these functions may be affected, leading the body to a pro-inflammatory pattern, which may affect the proper functioning of many tissues (Wellen & Hotamisligil, 2005). It is believed that the inflammation induced by obesity contributes to the development of many chronic diseases, including type 2 diabetes mellitus, atherosclerosis, liver disease and some forms of cancer (Lumeng & Saltiel, 2011; Gregor & Hotamisligil, 2011; Jung & Choi, 2014).

The study of the mechanisms by which obesity induces physiological disorders may be facilitated by the use of animal models, in particular rodents (Panchal & Brown, 2011; Nilsson et al., 2012). Currently, numerous experimental models of obesity are described in the literature and the choice of the best model depends on the objective of the study. The major ones used are the genetically or alimentary models. Genetic alterations can be classified as monogenic and are due to peripheral tissues mutations. For example, leptin-deficient (ob/ob) and leptin receptor deficient (db/db) mice have been used for studies of obesity and type II diabetes, demonstrating insulin resistance, hyperleptinemia, hyperphagia and extremely high plasma glucose levels at 6–10 weeks old (Zhang et al., 1994). These models are particularly important once there are homologous genes in man (Marques-Lopes et al., 2004). Another animal model that presents genetic alterations in the leptin receptor gene is the obese Zucker rat (fa/fa) (Phillips et al., 1996). This mouse is hyperphagic and presents reduced energy expenditure, preferential deposition of lipids in adipose tissue leading to the development of pronounced obesity at an early stage of life (White & Martin, 1997). However, these animals have normal glycemic levels and do not develop evident diabetes (Artiñano & Castro, 2009). In addition, another widely used model is the mice with a mutation in the chromosome that synthesizes the peptide related to the agouti protein (AGRP), a protein that acts on both the central nervous system (CNS) and tissues in the periphery to induce the obesity syndrome. In the CNS, agouti antagonize neural melanocortin receptor, resulting in obesity, hyperphagia and hyperinsulinemia while in the periphery, agouti expression in adipose tissue results in significant weight gains in mice (Michaud et al., 1997).

Monogenic animal models of obesity are useful because their obesity and adiposity are often severe resulting in a distinct phenotype, which may be extremely relevant to certain aspects of obesity research, such as the evaluation of drug effects (Hinney, Vogel & Hebebrand, 2010). Despite its etiology, the number of obese individuals in the world is increasing, and it is suggested that environmental or behavioral factors, such as excess amount of caloric food and lack of exercise activities, are the main contributors to the epidemic, rather than genetic changes (Buettner, Scholmerich & Bollheimer, 2007). Thus, whereas the experimental model should be as close as possible to the genesis of study, polygenetic animal models with diet-induced obesity have been preferably used in place of monogenetic models (Rosini, Silva & Moraes, 2012).

Obesity induced by diet in rodents can be obtained by different ways, with a great variation both in relation to the diet content and the rodent species used (West, Waguespack & McCollister, 1995). Most rodents tend to become obese on high-fat diets, but there are variable responses in weight gain, glucose tolerance, insulin resistance, triglycerides and other parameters depending on the strain. The C57B16/J mice are considered prone to obesity, and are one of the most commonly used rodent strain for diet-induced obesity, presenting pronounced increase in weight, as well as hyperinsulinemia and also hyperglycemia (Panchal & Brown, 2011). C57BL/6JOlaHsd mice, a strain of mice genetically prone to develop obesity and insulin resistance, showed a significant reduction of body weight and an improvement in insulin sensitivity after the transition from high to normal fat diet (Lombardo et al., 2016).

Other rodents, such as Wistar and Sprague-Dawley rats, are also susceptible to diet-induced obesity and popularly used in research as they readily gain weight on high-fat diets (Rosini, Silva & Moraes, 2012). The Sprague-Dawley rats, especially, have been studied for their ability to show a variable response to a high-fat diet, once some animals rapidly gain weight while others gain only as much weight as those fed a low-fat diet (Madsen et al., 2010), allowing for the study of animals that are naturally prone and resistant to obesity. Many studies have attempted to induce obesity in laboratory animals increasing only food intake, which would leads to weight gain and obesity. However, increasing dietary density or increasing the calorie intake of certain macronutrients, such as fats and carbohydrates, resulting in the total caloric intake ingested, is more efficient to trigger the disease. High-carbohydrate diets have been used to develop obesity in rats, but the association between carbohydrates and fats, or just fats, has shown more deleterious effects on animals (Sato-Mito et al., 2009). These diets rich in both fat and carbohydrates (fructose or sucrose), closely mimic the human diet, resulting in a similar effect on body weight, abdominal fat, hyperinsulinemia and hyperglycemia, leading to the metabolic syndrome (Surwit et al., 1995; Sato-Mito et al., 2009). It is important to note that the dietary fat source may result in small differences in phenotypes, since more pronounced manifestations of obesity and insulin resistance are observed when the source of fat contains substantial amounts of saturated fat and monounsaturated fat; such as lard, for example (Krishnan & Cooper, 2014).

Due to their size, ease of handling, and similarity to patterns found in humans (Eckmann, 2003; Araújo et al., 2008), the gerbil (*Meriones unguiculatus*) is a model already widespread in many parts of the world and well established for the study of various diseases such as giardiasis (Ventura et al., 2013), *Helicobacter pylori* infection and stomach cancer (Kodama, Murakami & Fujioka, 2004; Júnior et al., 2016), hearing disorders (Abbas & Rivolta, 2015), and more recently, as a good model for the study of visceralization of *Leishmania major* (Bakırcı et al., 2015). Li et al. (2016), selectively bred a group of spontaneously hyperglycemic diabetic *Mongolian gerbils* and established a spontaneous diabetic gerbil line with insulin resistance and leptin resistance as well as decreased adiponectin level in the serum. The occurrence of spontaneously obesity in the *Meriones unguiculatus* species of gerbils was reported several decades ago, when researchers observed that 10% of the animals became obese and showed metabolic alterations, even when fed standard diet (Vincent, Rodrick & Sodeman jr, 1979). Other species of desert rats, or gerbils, may also develop obesity when their typical diet is changed to standard rodent chow, such as *Psammomys obesus* (Khalkhal et al., 2012). Some studies addressed metabolic and immunological disorders related to obesity in this model of naturally obese animals (Xu, Liu & Wang, 2011; Liu, Xu & Wang, 2012). Semiane et al., (2016) found that the consumption of a high-carbohydrate diet induces metabolic disorders and damaged liver, that characterize nonalcoholic steatohepatitis in a desert gerbil, *Gerbillus gerbillus*, suggesting that this rodent represents a model for human metabolic diseases. Of note, there are no studies that induced obesity by a high fat and simple carbohydrates diet in the species *Meriones unguiculatus*, comparing them with animals fed on standard laboratory chow diet. Thus, we sought to investigate the metabolic changes in gerbils, resulting from a diet rich in fats and simple sugars, which is closer to dietary changes of modern occidental diet.

## Materials & Methods

Fourteen adult male (average age of 20 weeks, 60–80 g of weight) gerbils (*Meriones unguiculatus*) were obtained from the Animal Care Center of the Institute of Biological Sciences (ICB) of the Federal University of Minas Gerais (UFMG) and kept in the animal facility of the Department of Parasitology. After a week of acclimatization, the animals were randomly divided according to the weight into two groups of seven animals receiving standard diet (calorie density 4,15 kcal/g—CT group) or hypercaloric diet (calorie density 5,86 kcal/g—group OB). Dietary fatty acid composition was calculated based on data from Silva & Gioielli (2006), as shown in Table 1. The animals were maintained throughout the experimental period in two collective cages, with seven animals each, under controlled temperature and lighting, with free access to filtered water and diet. After 11 weeks of the experiment, and 12 h fasting, the gerbils were anesthetized (Ketamine (Agener União) 100 mg/kg and Xylazine (König) 12 mg/kg, i.p.) and blood was collected from the axillary artery. Blood samples were centrifuged at 12,000 ×g (3,500 rpm) for 10 min and the sera were used for biochemical analysis. Animals were euthanized and visceral adipose tissue (perirenal, epididymal and mesenteric) and liver were collected. All experiments were conducted in accordance with the guidelines of the Ethics Committee on Animal Use (CEUA) of UFMG (protocol 136/13).

**Table 1:** Composition of experimental diets.

Ingredients	(g/kg)	
	Standard	Hyper
*Casein*	200	200
*Corn starch*	**397.5**	**62**
*Sucrose*	**100**	**–**
*Maltodextrin*	**132**	**–**
*Gooseberry syrup*	–	**310**
*Lard*	–	**355**
*Soybean oil*	**70**	**20**
*Powdered cellulose*	50	50
*Mineral Mix (AIN-93G)*	35	35
*Vitamin Mix (AIN-93G)*	10	10
*Choline bitartrate*	2.5	2.5
*t-butylhydroquinone*	0.014	0.014
*Methionine*	3	3
**(kcal/g)**	**4.15**	**5.86**
Fatty acid composition, g/100 g^*^		
MUFA	22.10	42.32
PUFA	60.74	15.15
SFA	16.93	42.51
UFA/SFA	**4.89**	**1.35**

**Notes.**

TITLE MUFA monounsaturated fatty acid PUFA polyunsaturated fatty acid SFA saturated fatty acid UFA unsaturated fatty acid

* Silva & Gioielli, 2006.

### Assessment of energy intake, fatty acid intake, body weight and adiposity

Individual body weight was recorded weekly. Total intake of food was measured once a week, and total energy was calculated. Considering the composition of lard and soybean oil, the amounts of saturated, monounsaturated and polyunsaturated fatty acid intake was also calculated. At the end of the experiment, the relative weights of liver and epididymal adipose tissue were calculated (tissue weight/final body weight ×100).

### Dosages of cytokines and hormones in adipose tissue

Fragments of 100 mg of the epididymal adipose tissue were homogenized in protease inhibitor solution. Enzyme linked immunosorbent assays (ELISA) of the cytokines IL-6 (catalog number DY406-05), IL-10 (catalog number DY417-05), TFN-*α* (catalog number DY410-05), adiponectin (catalog number DY1119) and leptin (catalog number DY398-05), were performed from portion of the supernatant following the protocol recommended by the manufacturer (R&D Systems, Minneapolis, MN, USA).

### Dosages of total cholesterol, triglycerides and plasma glucose

All measurements were performed by enzymatic colorimetric assay (Labtest kit, Lagoa Santa, Brazil) as recommended by the manufacturer’s protocols.

### Extraction and quantification of hepatic lipids

The determination of a hepatic lipid profile was taken after extraction of the total lipids in organic solvent, as described by Folch, Lees & Stanley (1957). 100 mg of liver tissue were used. For determination of the total cholesterol and triglyceride levels, lipid extracts were resuspended in 500 µL of isopropanol, and dosage was performed by colorimetric enzymatic assay (Labtest kit, Lagoa Santa, Brazil).

### Histological analysis

Liver fragments were collected and washed with a saline solution. Tissues were then fixed in 10% buffered formalin, embedded in paraffin, and then sliced to a thickness of 4 µm and stained with Hematoxylin & Eosin (H&E). Representative areas of 30 images of liver tissue were calculated by the KS400 software on a Carl Zeiss image analyzer to evaluate hepatic steatosis.

### Evaluation of oxidative stress in the liver

Fragments of tissue (approximately 100 mg) were homogenized in saline solution and the supernatant was performed with the following dosages:

#### Dosage of antioxidant enzyme catalase

Catalase activity was determined as described by Nelson & Kiesow (1972). The decomposition of H_2_O_2_ due to catalase activity was assessed by the decrease in the absorbance of H_2_O_2_ at 240 nm. The catalase activity was expressed as moles of H_2_O_2_ utilized/min/g of liver proteins.

#### Dosage of antioxidant enzyme SOD

Superoxide dismutase (SOD) activity was based on its ability to scavenge superoxide (O${}_{2}^{-}$) radicals, decreasing the rate of auto-oxidation of pirogallol, according to the method of Dieterich et al. (2000). The enzyme activity was measured by monitoring the rate of decrease in optical density at 420 nm. The result was expressed as SOD units/ g of liver proteins. One unit of SOD is defined as the amount of enzyme that inhibits autoxidation of pyrogallol by 50%.

### Data analysis

To verify the distribution of the data the Shapiro–Wilk test was used, and to compare the means or medians the Student *t*-test or Mann Whitney was used, respectively. All statistical analyzes were performed using the program Prism (GraphPad Software, San Diego CA, USA), version 6.0. Significance level was considered at 5%.

## Results

**Figure 1:**
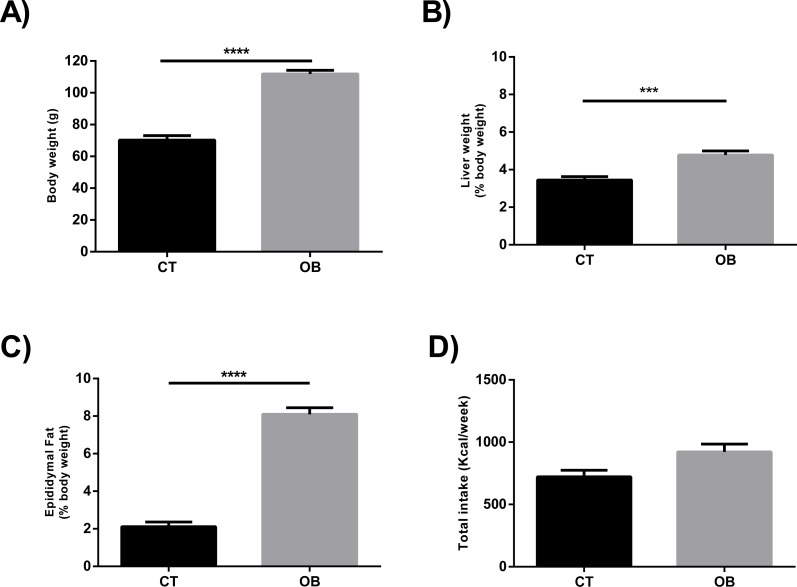
(A) Total body weight, ratio of (B) Liver (% body weight) and (C) Epididymal fat (% body weight), and (D) Total caloric intake (Kcal/week) in gerbil fed control (CT) or hyper (OB) diets during 11 weeks. Data were represented as means ± S.E.M. *n* = 7 per group. Statistical significance was indicated as (***) for *p* < 0.001 or (****) for *p* < 0.0001. *T*-test used for all data.

The Hyper diet contained higher proportions of total SFA and MUFA, and consequently lower ratio UFA/SFA, than the Standard diet (Table 1). In contrast to animals fed regular balanced chow (CT), we observed increased body weight (Fig. 1A) and relative liver weight (Fig. 1B) in animals fed Hyper diet (OB group). These results were supported by the increased adiposity (Fig. 1C), independently of the energy intake (Fig. 1D).

**Figure 2:**
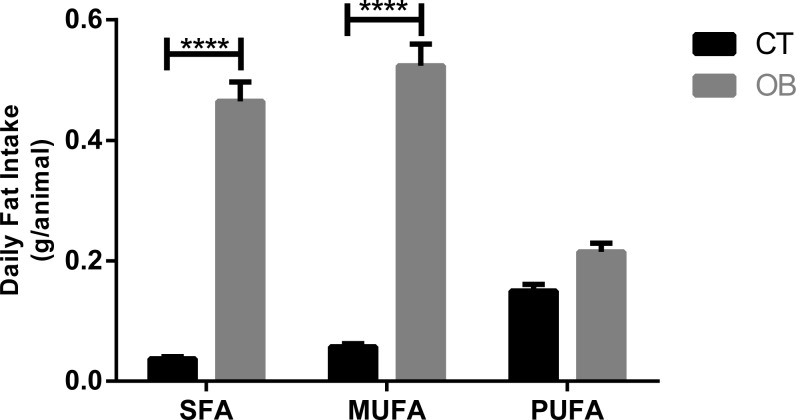
Fatty acid intake in gerbil fed control (CT) or hyper (OB) diets during 11 weeks. Data were represented as means ± S.E.M. *n* = 7 per group. Statistical significance was indicated as (****) for *p* < 0.0001. *T*-test used for all data. SFA, saturated fatty acid, MUFA, monounsaturated fatty acid, PUFA, polyunsaturated fatty acid.

Saturated and monounsaturated fatty acid intake (Fig. 2) was significantly higher (*p* < 0.0001) in animals fed with Hyper diet (OB group).

The animals with excess body weight also presented metabolic alterations characterized by increased blood glucose levels, increased circulating triglycerides and ectopic fat deposition in the liver (Table 2).

**Table 2:** Fasting glucose, blood lipid profile and ectopic (liver) lipid concentration in gerbil fed Control (CT) or Hiper (OB) diet after 11 weeks.

*Evaluated parameters*	CT	OB
*Fasting glucose (mg/dL)*	133.5 ± 3.26	182.0 ± 14.17*
*Blood cholesterol (mg/dL)*	82.75 ± 2.91	89.08 ± 3.45
*Blood triacylglycerol (mg/dL)*	180.2 ± 14.49	240.4 ± 16.61*
*Liver lipids (mg/g)*	52.43 ± 3.66	210.0 ± 16.45****
*Liver cholesterol (mg/g)*	4.39 ± 0.28	7.76 ± 0.57***
*Liver triacylglycerol (mg/g)*	3.28 ± 0.39	93.89 ± 5.90**

**Notes.**

Data were represented as means ± S.E.M. *n* = 7 per group. Statistical significance was indicated as (*) for *p* < 0.05, (**) for *p* < 0.01, (***) for *p* < 0.001 or (****) for *p* < 0.0001. Mann–Whitney test used in Fasting glucose and Liver triacylglycerol *T*-test used in Blood cholesterol, Blood triacylglycerol, Liver lipids and Liver cholesterol.

**Figure 3:**
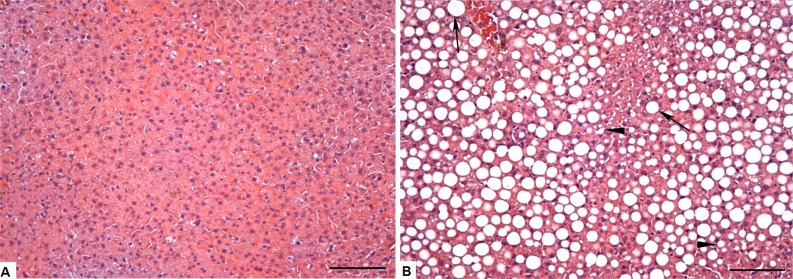
Photomicrograph of the liver tissue CT and OB groups (A) Control group showing normal liver parenchyma. (B) Obese group showing microvesicular (arrowheads) steatosis, but mainly the presence of large negative cytoplasmic vacuoles (arrow) that pushes the nucleus to the periphery of the hepatocyte, increases its size and compress the sinusoid capillaries. Bar = 100 µm.

**Figure 4:**
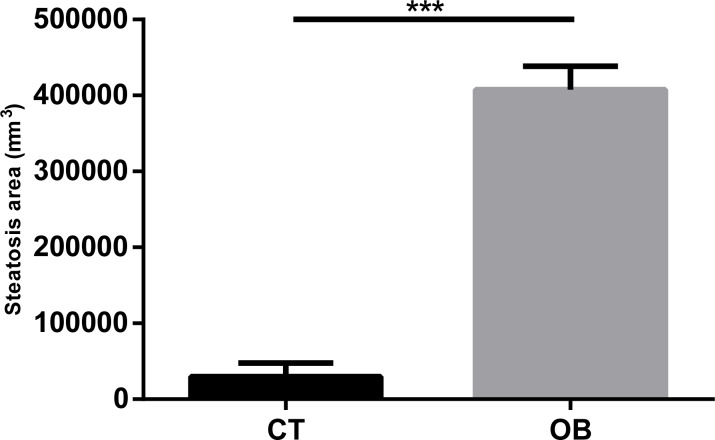
Morphometric analysis of steatosis area in liver tissue of Control and Obese gerbils after 11 weeks of Hyper diet. Data were represented as means ± S.E.M. *n* = 7 per group. Statistical significance was indicated as (***) for *p* < 0.001 by Mann–Whitney test.

We can clearly observe intense micro and macrovesicular hepatic steatosis in obese animals, as shown in Figs. 3A and 3B. These changes were quantitated by morphometric analysis (Fig. 4), with greater levels of fat deposition in the liver of the animals in the group OB by steatosis, compared to the control group (40,7811 ± 30,903 µm^2^ vs 29,906 ± 18,009 µm^2^, respectively).

Obese animals also showed significant reduction in the antioxidant capacity of the liver catalase enzymes (6.76 ± 0.40 vs. 4.65  ± 0.40 U/g of protein, *p* < 0.01) and superoxide dismutase (1.74 ± 0.07 vs. 1.40 ± 0.11 U/g of protein, *p* < 0.05) when compared to animals in the CT group.

**Figure 5:**
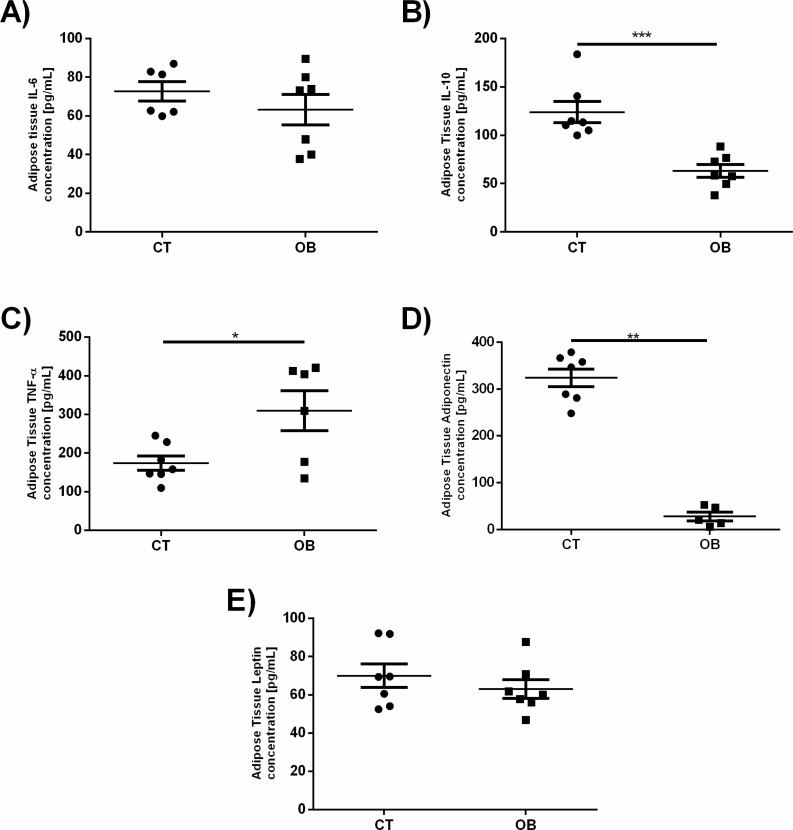
Adipose tissue concentration of the adipokines (A) IL-6, (B) IL-10, (C) TNF-*α*, (D) Adiponectin and (E) Leptin. Data were represented as means ± S.E.M. *n* = 7 per group. Statistical significance was indicated as (***) for *p* < 0.001 or (****) for *p* < 0.0001. Mann–Whitney test used in IL-6, IL-10 and Adiponectin. *T*-test used in TNF-*α* and Leptin.

Given that a number of changes in the pro-inflammatory and regulatory levels of cytokines and hormones are reported in obesity, as increased levels of TNF-*α*, IL-6 and leptin as well as reduction of IL-10 and adiponectin, we investigated whether such changes would be found in fat animal model proposed in this study (Fig. 5). Animals treated with a diet rich in fat and carbohydrates had increased TNF-*α* concentrations (OB =309.7 ± 51.71 pg/mL versus CT =173.8 ± 18.28 pg/ml) and reduction of IL-10 (OB =63.11 ± 6.53 pg/ml versus CT =123.9 ± 11.08 pg/ml) and adiponectin (OB =27.7 ± 9.23 pg/mL versus CT =324.1 ±  19.06 pg/ml) levels in adipose tissue when compared to animals fed control diet. IL-6 and leptin concentrations remained unchanged between groups.

## Discussion

Diets rich in simple sugars and fats generally cause increased body weight, and increased number and volume of adipocytes (Hauner, 2005). There is a deregulation of secretory activity of the adipose tissue, development of metabolic diseases, altered inflammatory responses and metabolism of lipids and glucose (Lumeng & Saltiel, 2011).

The purpose of this study was to investigate the metabolic changes in gerbils (*Meriones unguiculatus*) fed with a diet rich in fats and simple sugars. The present data demonstrate that animals developed obesity and exhibited decreased oxidative capacity, increased circulating glucose and triglycerides levels, high levels of hepatic total fat, cholesterol and triglyceride, contributing to the development of steatosis, and misbalance in pro-inflammatory/regulatory status in adipose tissue.

The Hyper diet was rich in mono and saturated fatty acids, with a low ratio of saturated/unsaturated fatty acid. Surprisingly, our results showed that although there was no difference in the total caloric intake, which implicates that the type of dietary fat is a primary obesogenic factor, as suggested previously (Crescenzo et al., 2015). PUFA are preferentially *β*-oxidized and inhibits de novo hepatic fatty acid synthesis and lipogenic gene expression, decreasing abdominal fat content and peripheral insulin resistance, compared with SFA (Bjermo et al., 2012; Moussavi, Gavino & Receveur, 2008). Fat oxidation is higher with an increase in polyunsaturated/saturated ratio intake (Moussavi, Gavino & Receveur, 2008). It has been shown that diet rich in unsaturated fatty acids could promote the proliferation of brown adipose tissue growth, as well as uncoupling protein-1 upregulation (Bargut, Aguila & Mandarim-de-Lacerda, 2016). That’s why diets rich in unsaturated fatty acids are considered less deleterious for human health than those rich in saturated fat (Crescenzo et al., 2015).

In the early stages of adipocyte hypertrophy, a series of phenomena such as oxidative stress, hypoxia and downregulation of some chemokines initiate the inflammatory process in this tissue (Bost et al., 2005; Ito et al., 2007). During obesity, there is also recruitment and activation of other immune cells such as macrophages which undergoes a phenotype polarization from anti-inflammatory profile of M2 macrophages or “alternatively activated” to the pro-inflammatory profile of M1 macrophages or “classically activated,” increasing the secretion of various proinflammatory cytokines, such as TNF-*α* and IL-6 and decreasing the production of anti-inflammatory factors such as adiponectin and IL-10, promoting a pro-inflammatory status in the host (Lumeng, Bodzin & Saltiel, 2007; Gregor & Hotamisligil, 2011; Jung & Choi, 2014; van Stijn et al., 2015).

TNF-*α* derived from macrophages induces the release of free fatty acids (FFA) from adipocytes via lipolysis (Cawthorn & Sethi, 2008) and at the same time, these FFA released, strongly stimulate TNF-*α* production by macrophages (Klop, Elte & Cabezas, 2013). This paracrine interaction between adipocytes and macrophages is a “vicious cycle”, further accelerating the adipose tissue inflammation and promoting inhibitory effects on energy metabolism (Clemente-Postigo et al., 2011). In addition, the TNF-*α* produced by adipose tissue expansion, can induce the increase of synthesis and release of leptin (Paz-Filho et al., 2012), which is involved in the regulation of food intake and energy expenditure (St-Pierre & Tremblay, 2012). Circulating levels of leptin and its expression in adipose tissue are increased in obese individuals, probably because of leptin resistance (Myers Jr et al., 2010).

Increased values of TNF-*α* were observed in our obesity model. However, we found no significant change in the amount of IL-6 or leptin in obese animals when compared to the controls. It is known that in humans, leptin production by adipose tissue is influenced by paracrine IL-6 production (Trujillo et al., 2004). Furthermore, it has been also reported that the concentration of leptin in relation to adipose mass in humans, decreases as factors associated with metabolic syndrome worsen, especially in hypertriglyceridaemia (Paz-Filho et al., 2009). The unbalanced production of cytokines, with a reduced production of anti-inflammatory cytokines, such as adiponectin, is a characteristic of a diet-induced obesity. Under normal conditions, this cytokine is widely produced by adipose tissue and acts mainly promoting the activation of protein kinase activated by adenosine monophosphate (AMPK), which phosphorylates regulatory enzymes of glycolysis, gluconeogenesis and lipid oxidation in liver and skeletal muscle (Van Stijn et al., 2015). Another important function of adiponectin is the induction of IL-10 expression in M2 macrophages, an important regulatory cytokine (Van Stijn et al., 2015). In obese individuals, plasma levels of IL-10 can be re-established with weight loss (Golubović et al., 2013). Diet-induced gerbil obesity model showed decreased levels of adiponectin and IL-10 in adipose tissue, suggesting an association between metabolic disorders found in plasma and liver and the altered levels of these cytokines.

Increased levels of TNF-*α* production and lower levels of adiponectin are associated with peripheral resistance to insulin (Yamauchi et al., 2002), which can be observed by an increase in plasma glucose in OB animals. The hypertriglyceridemia observed in this model may result from increased release of FFA from adipocytes following activation of the lipase hormone sensitive enzyme by TNF-*α* (Clemente-Postigo et al., 2011) associated with lower oxidation of FFA in adipose tissue and less use of glucose in the liver and skeletal muscle. There is also less activation of the lipoprotein lipase (LPL) enzyme, thus causing lower uptake of very low density lipoproteins (VLDL) and increased levels of circulating triglycerides (Yamauchi et al., 2002). Once the OB animals showed decreased adiponectin concentration in adipose tissue and increased TNF-*α*, we believe that these metabolic pathways are certainly impaired, as it has been reported in humans (Matsubara, Maruoka & Katayose, 2002; Chang et al., 2012).

Steatosis is often an initial trigger for a series of diseases in the liver tissue, such as non-alcoholic steatohepatitis (NASH) and cirrhosis (Tilg & Moschen, 2010). In steatosis, hepatocytes are more susceptible to the action of bacterial toxins derived from the intestine, mitochondrial dysfunction, dysregulated apoptosis, oxidative stress, the action of pro-inflammatory cytokines and adipokines, and activation of pro-fibrogenic factors, that lead to disease progression (Tarantino, Savastano & Colao, 2010; Polyzos et al., 2012).

Under normal circumstances, the liver aerobic metabolism involves a stable production of pro-oxidants such as reactive oxygen species (ROS) and reactive nitrogen species (RNS), which are equilibrated by a similar rate of its consumption by antioxidants (Winterbourn, 2008). An imbalance in these pathways, in favor of oxidative stress, characterized by oxidation of essential biomolecules by ROS and RNS, can cause the loss of important cellular biological functions and compromise cell viability (Görlach et al., 2015). In addition, ROS may indirectly activate transcription factors such as nuclear factor kB (NF-kB) (Tornatore et al., 2012) increasing the production of cytotoxic mediators, pro-inflammatory and fibrogenic mediators by the Kupffer cells (Takeuchi-Yorimoto et al., 2013). As the hepatic disease progresses, there is further reduction of antioxidant capacity of the liver, as demonstrated by the significant reduction in activity of catalase and superoxide dismutase, and is exacerbated further in the later stages of steatohepatitis, as demonstrated in a study of patients with NASH (Videla et al., 2004). Thus, we consider the reduction of hepatic activity of antioxidant catalase enzymes and superoxide dismutase, observed in this work, may suggest a transitional state between NAFLD and NASH, and it is possible that, over time, the increased oxidative stress in the liver from animals in the OB group may lead to serious manifestations of liver disease.

## Conclusions

Hypercaloric diets with high fat and simple carbohydrates leads to weight gain, systemic metabolic alterations, adipose tissue inflammation, hepatic steatosis, and oxidative stress in gerbils (*Meriones unguiculatus*). This model can be widely adopted as an effective model for the study of obesity induced by diet, helping scientists design studies with tightly controlled conditions and therefore improve our understanding of obesity and related diseases.

##  Supplemental Information

10.7717/peerj.2967/supp-1 Data S1 Raw data

## Additional Information and Declarations

### Data Availability

The following information was supplied regarding data availability: The raw data has been supplied as a Data S1.
