# Supplementary material for: Obesity-induced diet leads to weight gain, systemic metabolic alterations, adipose tissue inflammation, hepatic steatosis, and oxidative stress in gerbils (Meriones unguiculatus)
Source: PeerJ. 2017 Mar 2;5:e2967. doi: 10.7717/peerj.2967 (PMC5337087; doi:10.7717/peerj.2967)
Supplement: Data S1 [file peerj-05-2967-s001.pdf]

RAW DATA - **Gerbils (*Meriones unguiculatus*) as a new experimental model of obesity induced by diet**

			PARAMETERS OF WEIGHT			
groups	inicial weight	animals	weight 10ª week	final weight	adipose tissue weight (%)	liver weight (%)
CT	67.8	CT1	75	72	1.75	3.625
	61.4	CT2	57	58	1.206897	3.293103
	68	CT3	72	70	1.942857	2.714286
	70	CT4	75	69	2.942029	3.289855
	82	CT5	79	74	1.945946	3.405406
	72.3	CT6	71	67	1.985075	3.462687
	77.8	CT7	83	82	3.036585	4.341464
OB	81.6	OB1	112	116	7.448276	4.008621
	70	OB2	121	118	8.70339	5.245763
	66	OB3	109	114	9.298245	4.412281
	70	OB4	110	111	8.900901	4.945946
	66	OB5	110	113	8.168141	5.292036
	68	OB6	108	112	7.401786	4.098214
	60.5	OB7	100	99	6.747475	5.424242

weeks	Food ingestion CT	OB
1	327.03	322.91
2	225	265
3	144.8	145.8
4	228.6	240
5	372.2	256.7
6	214.8	227
7	251	199
8	268.9	247.3
9	234	224
10	255	153

groups	animals	biochemical PARAMETERS					
		glucose	cholesterol	triglycerides	liver fat	liver cholesterol	liver triglycerides
CT	CT1	138.9863892	89.19963755	183.6437886	37	4.037195908	2.788976205
	CT2	128.2831791	77.45445479	174.9432195	66	5.365673642	2.450812108
	CT3	121.1245372	72.0249633	127.0476582	53	4.425460004	2.249836727
	CT4	133.2615123	75.31514166	172.8761213	45	4.018310612	4.199459778
	CT5	135.3905176	90.97972486	154.855879	55	5.186740413	4.547920749
	CT6	143.7679151	85.05763211	196.909591	50	4.450851072	3.458123917
	CT7	218.3724812	89.19580603	250.7852988	61	3.247445495	15.83*
OB	OB1	125.7048564	83.80530851	208.883802	141	5.246810896	70.4453105
	OB2	218.0872054	106.2417878	178.2016606	235	7.874916246	105.9254763
	OB3	185.9263567	83.93802994	306.8713391	209	6.677745851	93.57167703
	OB4	235.1000675	94.34969896	265.7781736	182	8.841027987	100.8857938
	OB5	156.468798	78.9567347	255.7584851	244	9.779371972	104.9770707
	OB6	190.6100058	91.17462687	259.4691581	189	7.377345119	73.76068376
	OB7	162.0067531	85.0913076	207.6928596	270	8.503543449	107.6772936

		oxidative stress		
groups	animals	SOD liver	CATALASE liver	TBARS liver
CT	CT1	1.910335242	12.68*	0.54
	CT2	1.97636402	8.19425	0.35
	CT3	1.810151974	7.172943038	0.71
	CT4	1.841864991	6.701639344	0.4
	CT5	1.701750184	6.355152514	0.54
	CT6	1.641676941	6.923169268	0.8
	CT7	1.439865119	5.21507159	0.36
OB	OB1	0.916726891	3.414819588	0.16
	OB2	1.26946618	5.259111901	2.1
	OB3	1.430178701	4.385751806	0.16
	OB4	1.290803992	5.140137615	0.27
	OB5	1.94029077	6.148038361	3.13*
	OB6	1.494661898	5.118554688	1
	OB7	1.487207833	3.140429564	0.67

		steatosis	IMMUNOLOGICAL PARAMETERS - adipose TISSUE				
groups	animals	total area	IL6 TEC ADIP	IL10 TEC ADIP	TNF TEC ADIP	ADIPONECTIN	LEPTIN
CT	CT1	0	*233.77**	114.74	245.35	378.75	92.18
	CT2	23030.96	62.7	140.36	228.48	366.49	91.86
	CT3	61573.16	59.84	99.9	182.52	358.04	60.51
	CT4	0	81.42	113.39	158.28	346.97	69.4
	CT5	0	62.13	183.71	146.89	289.25	54
	CT6	0	86.99	105.02	145.63	248.24	69.51
	CT7	124740.18	82.85	110.42	109.79	281.29	52.48
OB	OB1	284262.2	39.98	57.59	412.74	13.52	57.79
	OB2	490385.47	73.13	58.66	*134.45**	5.8	70.81
	OB3	422669.58	79.99	76.82	*117.17**	46.36	61.7
	OB4	325958.97	73.99	49.61	420.75	52.57	60.07
	OB5	454948.16	89.57	72.67	404.31	20.22	87.62
	OB6	378920.89	37.69	37.94	309.02	*497.77**	55.95
	OB7	497531.3	47.84	88.48	177.04	0	46.84
